# Changes in socioeconomic inequalities in food consumption among Brazilian adults in a 10-years period

**DOI:** 10.3389/fnut.2022.1020987

**Published:** 2022-12-15

**Authors:** Maria Laura da Costa Louzada, Janaína Calu Costa, Caroline dos Santos Costa, Andrea Wendt, Catarina Machado Azeredo

**Affiliations:** ^1^Center of Epidemiological Studies in Nutrition and Health (NUPENS), University of São Paulo, São Paulo, Brazil; ^2^School of Public Health, Department of Nutrition, University of São Paulo, São Paulo, Brazil; ^3^International Center for Equity in Health, University of Pelotas, Pelotas, Brazil; ^4^Postgraduate Program in Health Technology, Catholic University of Paraná, Curitiba, Brazil; ^5^School of Medicine, Federal University of Uberlandia, Uberlandia, Brazil

**Keywords:** ultra-processed food, socioeconomic inequality, food consumption, time trend, survey

## Abstract

**Objective:**

To evaluate changes in socioeconomic inequalities in food consumption in Brazil over a 10-year period.

**Methods:**

Data on 24-h recalls of adults (aged 20 years or more) from the 2008/9 (*n* = 26,327) and 2017/8 (*n* = 37,689). Brazilian Dietary Survey were analyzed. We used the Nova classification system to group food items and estimate the percentage of total energy from ultra-processed foods and plant-based natural or minimally processed foods. For sex and area of residence, we calculated the percentage points (p.p.) difference between the estimates for women and men, and rural and urban populations. Negative values indicate higher consumption among men or urban residents, positive values indicate higher consumption among women or rural residents, and zero indicates equality. For education and wealth levels we calculated the slope index of inequality (SII). The SII varies from −100 to 100, with positive values indicating higher consumption among more educated or wealthiest groups, negative values indicating higher consumption among less educated or poorest groups, and zero equality.

**Results:**

Over the period, we observed a reduction in the percentage of total energy from plant-based natural/minimally processed foods from 13.0 to 12.2% and an increase in that of ultra-processed foods from 17.0 to 18.3%. The urban population and those in the wealthier and more educated groups presented higher consumption of ultra-processed foods and lower consumption of plant-based natural/minimally processed foods in both survey years. Over the 10-year period, there was an overall reduction of the socioeconomic inequalities, mainly explained by the greater increase in ultra-processed food consumption by the rural population and those from the poorest and less educated groups (difference for area −7.2 p.p. in 2008/9 and −5.9 p.p. in 2017/8; SII for education 17.7 p.p. in 2008/9 and 13.8 p.p. in 2017/8; SII for wealth 17.0 p.p. in 2008/9 and 11.2 p.p. in 2017/8).

**Conclusion:**

Socioeconomic inequalities in food consumption decreased in Brazil, but it may lead to the overall deterioration of the dietary quality of the more vulnerable groups.

## Introduction

Globally, food systems are experiencing rapid and drastic changes characterized by the reduction in the consumption of traditional meals based on natural or minimally processed foods and the increase in the consumption of highly processed ready-to-eat products (1, 2). These changes conflict with the recommendations of a diet that promotes human and planetary health, which comprises the avoidance of ultra-processed foods (3) and the consumption of a plant-based diet with a low to moderate amount of seafood and poultry and diverse combinations of fruits, vegetables, legumes, and whole grains (4). Consequently, losses are observed for nutrition, public health, and the environment, including increases in rates of obesity, diabetes, and cardiovascular diseases, in addition to increased carbon budgets, climate risks, and biodiversity impairment (5).

Food consumption is structurally conditioned by social inequality (6) and, therefore, not homogeneously distributed among individuals. Unequal access to economic resources, food supply and retail markets make those less economically privileged more vulnerable to a low-quality diet and an increased burden of its negative effects (7, 8). Thus, analyzing the global trend of food consumption may disguise differences among social groups over time.

The social gradient in food consumption in high-income countries shows a clear pattern of low-income individuals presenting a higher consumption of unhealthy food and lower consumption of healthy food compared to high-income individuals (8). In middle-income countries, some complex relations have been reported, with less educated individuals eating both less healthy food, such as fruits and vegetables, and unhealthy food, such as soft drinks, compared to those located upper in the social ladder (9, 10). A telephone-based study that evaluated the frequency of consumption of some food consumption markers of adults living in Brazilian state capitals only showed that, from 2008 to 2019, the consumption of fruits and vegetables was more frequent among those more educated. In the same study, it was observed an increase in educational inequality due to the increasing consumption of fruits and vegetables among those more educated, not followed by the less educated. On the other hand, the regular consumption of soft drinks was more frequent among those in the intermediate groups of education, and the educational inequality has decreased, due to a reduction in soft drinks consumption in all groups, especially among those more educated (9).

The assessment of inequality changes in food consumption over time is essential for health planning since it considers different patterns from population subsets, revealing groups in social disadvantage and consequently contributing to more effective interventions. Middle-income countries are of particular interest, due to their high social inequalities, limited resources, and high burden of non-communicable diseases. Moreover, the literature of food consumption inequality in middle-income countries tends to be limited to individuals living in highly urbanized areas, not reflecting the reality of the rural areas of the country and it has been based on limited food markers, which compromises the understanding of inequality in food consumption (11, 12). This reinforces the need to explore inequalities in different domains such as gender, education, area of residence and wealth in order to find the more vulnerable groups.

In the present study, we aimed to evaluate changes in socioeconomic inequalities in the consumption of ultra-processed and plant-based natural or minimally processed foods among Brazilian adults in a 10-years period.

## Methods

Data used in this study are from the individual food consumption modules of two editions of the Brazilian Household Budget Surveys (in Portuguese, *Pesquisa de Orçamentos Familiares*—POF) carried out by the Brazilian Institute of Geography and Statistics from May 2008 to May 2009 (hereafter called POF 2008) and from July 2017 to July 2018 (hereafter called POF 2017) (13, 14).

Both surveys used complex clustered sampling procedures in two stages, with geographic and statistical stratifications of the primary sampling units, which correspond to the sectors or clusters of sectors based on the Brazilian Demographic Census. In the first stage, primary sampling units were selected with probability proportional to the number of households in each sector by simple random sampling. The selected primary sampling units were distributed uniformly throughout the four trimesters of the study, in order to reproduce, within each stratum, the seasonal variation in income, prices, and food purchase and consumption. Then, permanent private households were selected using simple random sampling without replacement within each of the primary sample units selected.

For the individual food consumption module, subsamples of households were randomly selected from the original survey samples and corresponded to 24.3% of the full sample in 2008 and 34.7% in 2017. Food consumption data were collected for all residents aged ten years and over. The subsamples are representative of the Brazilian population living in private households. For this study, only data from adults aged 20 years and over were analyzed (26,327 individuals in 2008 and 37,689 in 2017).

Information on food consumption was collected using two 24-h food records in 2008 and two 24-h food recalls in 2017, both on non-consecutive days. In the food records, individuals detailed all foods and drinks consumed on the day in question (over 24 h) and the quantities of each item, referring to household measures. The food records were reviewed at the household by the interviewer together with the participant, typing the data in a program specially developed for this research. In the 24-h food recalls, the participants were asked, in personal interviews, about all the foods and drinks consumed on the day prior to the interview. Data collection was conducted following a structured script, in sequential stages of questioning the food, employing the Automated Multiple-Pass Method, using a software specifically designed for this assessment. To allow comparability between databases, some harmonization strategies were applied, including database compatibility and reanalysis of the information from the 2008 survey using the same food composition table applied in the 2017 survey (15).

### Food consumption

Food consumption variables were defined based on the NOVA food classification system (16): the percentage of total energy from ultra-processed foods (the unhealthy eating indicator) and the percentage of plant-based natural or minimally processed foods (the healthy eating indicator).

Ultra-processed foods include industrial formulations typically ready for consumption made of numerous ingredients, often obtained from high-yield crops, such as sugars and syrups, refined starches, oils and fats, and protein isolates, in addition to remains of intensive animal farming. These formulations are made to be visually attractive, have a seductive aroma, and very intense flavors, using sophisticated combinations of flavorings, dyes, emulsifiers, sweeteners, thickeners, and other additives that modify the sensory attributes. Examples are cookies, candies, salty snacks, soft drinks, artificial juices, and several ready-to-eat meals (16).

Natural or minimally processed foods are edible parts of plants or animals, mushrooms and algae, soon after their separation from nature or altered by industrial processes such as removal of inedible parts, dehydration, milling, pasteurization, freezing and other processes that do not involve the addition of other substances. Their main aim is to extend the life of grains (cereals), legumes (pulses), vegetables, fruits, nuts, milk, meat and other foods, enabling their storage for longer use, and often to make their preparation easier or more diverse. The set of plant-based natural or minimally processed food was defined based on international recommendations for a healthy and sustainable diet (4, 17, 18) and includes fruits (excluding juices), vegetables, legumes, nuts and seeds, and whole grains (excluding flour). In the analysis, both the composite indicator—based on the sum of the five food groups listed above—and the individual subgroups were used.

### Socioeconomic variables

To assess inequalities in food consumption between population subgroups, four socioeconomic and demographic variables were used: sex (female, male), area of residence (urban, rural), education level (none, 1–4, 5–8, 9–11, 12–13, 14 or more years of education), and wealth quintiles (Q1–Poorest to Q5–Wealthiest). Following the methodology employed in international household and health surveys, the wealth quintiles are based on the wealth index, a composite measure of living standards calculated using data on household's ownership of selected assets (such as TV, radio, shower, bed, computer, and vehicles), materials used for construction, type of water access and sanitation facilities, etc. It was generated using principal component analysis and, from the factors resulting from the model, individual households were placed on a continuous scale of relative wealth (19). These standardized scores were then ranked and divided into five equally-sized groups, the wealth quintiles, with the first quintile representing the poorest 20% in the sample and the fifth quintile representing the wealthiest 20%.

### Statistical analyses

We described the means and 95% confidence intervals (95% CI) for the food consumption indicators in 2008 and 2017 for the whole country. In order to describe inequalities according to each of the selected stratifiers, we presented the estimates using graphs called equiplots, which make it possible to visualize both the consumption estimates in each group and the distance between the categories, which represents absolute inequality. For the subgroups that are part of the set of plant-based natural or minimally processed foods, we described the mean and 95% CI for each survey according to the socioeconomic variables.

Simple and complex measures were used to address the magnitude of socioeconomic and demographic inequalities. For sex and area of residence, the binary variables, we calculated absolute inequality as the difference in percentage points (p.p.) between the estimates for women and men as well as for rural and urban areas. The estimates and respective 95% CI were obtained from linear regression models with men and urban area as reference categories, and its coefficient represents the gaps between the groups. Negative values indicate higher consumption among men, whereas positive values indicate higher consumption among women, and zero indicates equality. The same was obtained for area of residence, for which the estimate for urban area was subtracted from the estimate for rural area.

For education level and wealth quintiles, the ordinal variables, we calculated a complex measure to evaluate inequalities, the slope index of inequality (SII). This index is a measure of the difference in the outcomes between the top and the bottom groups, taking into account in its estimation the values for all intermediate categories as well as the size of each group. The index is the slope resulting from a linear regression model and expresses the absolute inequality in p.p.; it can vary from −100 to 100, with positive values indicating higher consumption among more educated or wealthiest groups, negative values indicating higher consumption among less educated or poorest groups, and zero indicating equality (20).

Evidence of differences in the estimates between the 2008 and the 2017 surveys was considered based on the non-overlapping of confidence intervals.

All analyses were performed in Stata 15^®^ (StataCorp. 2017. Stata Statistical Software: Release 15. College Station, TX: StataCorp LLC) using expansion factors and sample weights with the *svy* prefix command for survey data analysis.

## Results

### Consumption of ultra-processed foods and plant-based natural or minimally processed foods

**Figure 1:**
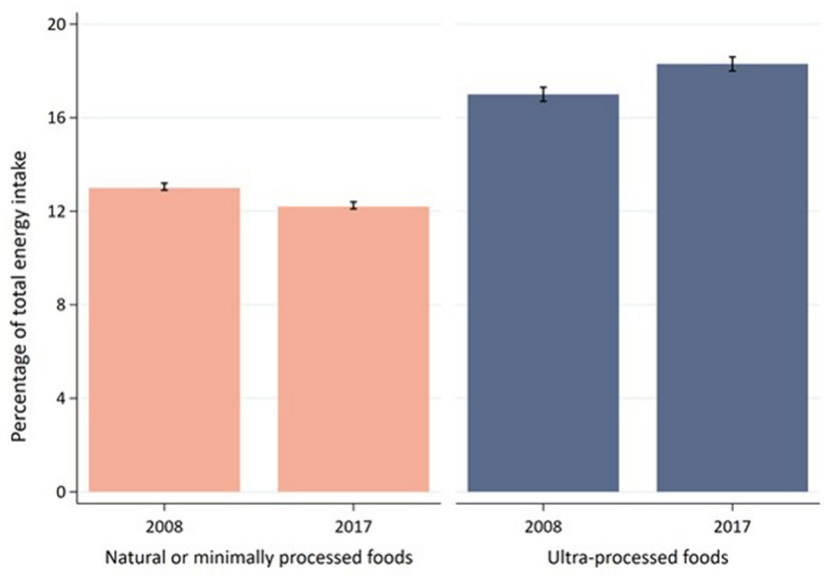
Percentage of total energy intake (mean and 95% confidence intervals) from the set of healthy foods and ultra-processed foods at the national level in 2008 and 2017 by Brazilian adults aged 20 years or more. Estimates for Healthy foods: 13.0, 95%CI 12.9; 13.2 (2008) and 12.2, 95%CI 12.1; 12.4 (2017); Estimates for Ultra-processed foods: 17.0, 95%CI 16.7; 17.3 (2008) and 18.3, 95%CI 18.0; 18.6 (2017). Evidence of differences in the estimates between the 2008 and the 2017 surveys was considered based upon the non-overlapping of confidence intervals.

Estimates of the consumption of plant-based natural or minimally processed foods and of ultra-processed foods in 2008 and 2017 for the whole group of Brazilian adults are presented in Figure 1. Between the two surveys, a reduction in the average caloric contribution of the set of plant-based natural or minimally processed foods was observed, decreasing from 13.0% in 2008 to 12.2% in 2017. On the other hand, the consumption of ultra-processed foods increased from 17.0% in 2008 to 18.3% of total energy intake in 2017.

**Figure 2:**
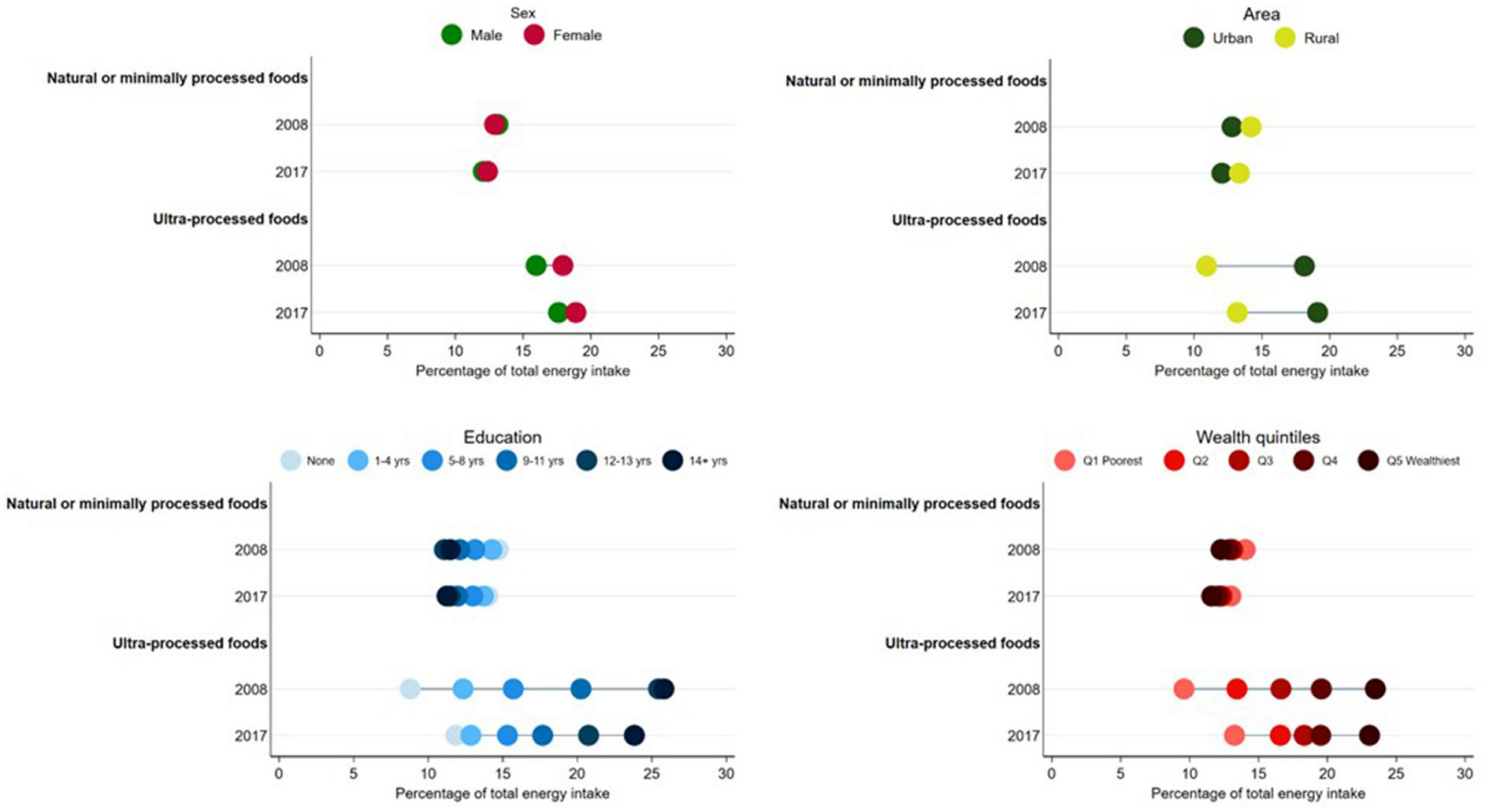
Percentage of total energy intake from the set of healthy foods and ultra-processed foods by sex, area of residence, education, and wealth quintiles in 2008 and 2017.

In Figure 2, we present estimates of consumption for the set of plant-based natural or minimally processed foods and ultra-processed foods in both surveys according to sex, area of residence, education, and wealth quintiles and, in Table 1, the inequality measures for both indicators, by the same socioeconomic and demographic variables.

**Table 1:** Inequality measures for the set of plant-based natural or minimally processed foods and ultra-processed foods in 2008 and 2017.

	**Difference**^a^ **(percentage points)**				**Slope index of inequality**^b^ **(percentage points)**			
	**2008**		**2017**		**2008**		**2017**	
	**Estimate**	**95%CI**	**Estimate**	**95%CI**	**Estimate**	**95%CI**	**Estimate**	**95%CI**
**Plant-based natural or minimally processed foods**								
Sex	−0.2	−0.5; 0.0	0.3	0.1; 0.5^c^	-	-	-	-
Area	1.4	1.0; 1.8	1.3	1.0; 1.6	-	-	-	-
Education	-	-	-	-	−3.9	−4.5; −3.4	−3.3	−3.7; −2.8
Wealth quintiles	-	-	-	-	−1.9	−2.5; −1.3	−1.5	−2.0; −1.0
**Ultra-processed foods**								
Sex	2.0	1.5; 2.5	1.3	0.9; 1.7	-	-	-	-
Area	−7.2	−7.8; −6.6	−5.9	−6.4; −5.4^c^	-	-	-	-
Education	-	-	-	-	17.7	16.7; 18.8	13.8	12.9; 14.6^c^
Wealth quintiles	-	-	-	-	17.0	15.9; 18.2	11.2	10.2; 12.2^c^

a Difference (female—male, rural—urban) estimated for binary stratifiers.

b Slope index of inequality estimated for ordinal stratifiers.

c Evidence of differences in the estimates between the 2008 and the 2017 surveys was considered based upon the non-overlapping of confidence intervals.

In 2008, the sex difference in the percentage of energy from the set of plant-based natural or minimally processed foods was −0.2 p.p., indicating a slightly higher consumption among men when compared to women. However, this pattern changed in 2017, as women presented a 0.3 p.p. higher consumption when compared to men. The caloric contribution of ultra-processed foods was higher for women in both surveys but there was a small reduction in the inequality in 2017 (difference of 2.0 p.p. in 2008 and 1.3 p.p. in 2017).

Differences between individuals living in urban and rural areas were small for the set of plant-based natural or minimally processed foods, with slightly higher consumption in the rural area in both 2008 and 2017 (difference in 2008 = 1.4 p.p.; difference in 2017 = 1.3 p.p.). An opposite pattern was observed for ultra-processed foods: their caloric contribution was higher for those living in urban areas in both surveys but there was a small reduction in the inequality in 2017 (difference of −7.2 p.p. in 2008 and −5.9 p.p. in 2017).

The consumption of the plant-based natural or minimally processed foods decreased as the education level increased in both surveys, indicated by negative values of SII. However, a small reduction in the inequality magnitude was observed between 2008 and 2017 (SII of −3.9 p.p. in 2008 and −3.3 p.p. in 2017). The opposite was observed for ultra-processed foods, for which the consumption was higher among individuals with higher education levels (SII in 2008 = 17.7 p.p.; SII in 2017 = 13.8 p.p.). Between 2008 and 2017, there was an increase in the consumption of ultra-processed foods among individuals with lower education levels (zero or 1–4 years of education) and a reduction among those with higher education levels (5–8 years, 9–11 years, 12–13 years, and 14 or more years) and, consequently, a reduction of the inequality.

Similarly, the consumption of the set of plant-based natural or minimally processed foods was inversely associated with wealth in both years and the magnitude of the inequality also slightly reduced (SII −1.9 p.p. in 2008 and −1.5 p.p. in 2017). Oppositely, the consumption of ultra-processed foods was higher among the wealthiest groups in comparison to the poorest. However, between 2008 and 2017, there was a reduction in the gap between the categories (SII of 17.0 p.p. in 2008 and 11.2 p.p. in 2017), due to an increase in the consumption of ultra-processed foods among the three more disadvantaged groups and a stabilization in the fourth and fifth quintiles.

### Consumption of fruits, vegetables, pulses, nuts and seeds, and whole grains

Estimates of the consumption of each subgroup from the set of plant-based natural or minimally processed foods in 2008 and 2017 are presented in Table 2. For Brazilian adults as a whole, we observed a reduction in the consumption of fruits (from 4.0% in 2008 to 3.4% in 2017) and pulses (from 7.1% in 2008 to 6.5% in 2017) and a slightly increase in the consumption of vegetables (1.5% in 2008 to 1.8% in 2017). No differences were observed in the consumption of whole grains (0.3% in both surveys) and nuts and seeds (0.1% in and 0.2% in 2017, presenting overlapping confidence intervals).

**Table 2:** Consumption of subgroups of the set of plant-based natural or minimally processed foods (as percentage of total energy intake) by sex, area of residence, education, and wealth quintiles in 2008 and 2017.

	**Fruits** **(% and 95%CI)**		**Vegetables** **(% and 95%CI)**		**Pulses** **(% and 95%CI)**		**Nuts and seeds** **(% and 95%CI)**		**Whole grains** **(% and 95%CI)**	
	**2008**	**2017**	**2008**	**2017**	**2008**	**2017**	**2008**	**2017**	**2008**	**2017**
**Sex**										
Male	3.4 (3.2; 3.5)	2.7 (2.7; 2.8)	1.5 (1.5; 1.5)	1.7 (1.7; 1.7)	7.9 (7.8; 8.1)	7.2 (7.0; 7.3)	0.1 (0.1; 0.2)	0.2 (0.2; 0.2)	0.2 (0.2; 0.3)	0.3 (0.2; 0.3)
Female	4.5 (4.4; 4.6)	4.0 (3.9; 4.1)	1.6 (1.6; 1.6)	1.9 (1.9; 1.9)	6.4 (6.2; 6.5)	5.9 (5.8; 6.0)	0.1 (0.1; 0.2)	0.3 (0.2; 0.3)	0.3 (0.2; 0.3)	0.3 (0.3; 0.4)
**Area of residence**										
Urban	4.0 (3.9; 4.1)	3.4 (3.3; 3.5)	1.6 (1.5; 1.6)	1.8 (1.8; 1.8)	6.9 (6.7; 7.0)	6.3 (6.2; 6.4)	0.1 (0.1; 0.2)	0.2 (0.2; 0.3)	0.2 (0.2; 0.3)	0.3 (0.3; 0.3)
Rural	3.9 (3.6; 4.1)	3.3 (3.1; 3.4)	1.5 (1.4; 1.5)	1.8 (1.7; 1.8)	8.4 (8.0; 8.8)	7.7 (7.5; 8.0)	0.1 (0.1; 0.2)	0.2 (0.2; 0.3)	0.3 (0.2; 0.4)	0.4 (0.3; 0.5)
**Education**										
None	3.7 (3.4; 3.9)	3.6 (3.3; 3.8)	1.6 (1.6; 1.7)	1.9 (1.8; 1.9)	9.1 (8.7; 9.4)	8.2 (7.9; 8.4)	0.0 (0.0; 0.1)	0.1 (0.1; 0.2)	0.3 (0.2; 0.4)	0.3 (0.2; 0.3)
1–4 years	3.9 (3.7; 4.1)	3.5 (3.3; 3.7)	1.6 (1.6; 1.7)	2.0 (1.9; 2.0)	8.4 (8.1; 8.6)	7.9 (7.6; 8.1)	0.1 (0.1; 0.1)	0.1 (0.1; 0.2)	0.3 (0.2; 0.3)	0.3 (0.2; 0.3)
5–8 years	3.7 (3.6; 3.9)	3.3 (3.2; 3.5)	1.5 (1.5; 1.6)	1.9 (1.8; 1.9)	7.6 (7.3; 7.8)	7.3 (7.2; 7.5)	0.1 (0.1; 0.1)	0.1 (0.1; 0.2)	0.2 (0.1; 0.2)	0.3 (0.2; 0.3)
9–11 years	3.9 (3.7; 4.0)	3.0 (2.8; 3.1)	1.5 (1.4; 1.5)	1.8 (1.7; 1.8)	6.4 (6.2; 6.6)	6.8 (6.6; 7.0)	0.2 (0.1; 0.2)	0.2 (0.1; 0.2)	0.2 (0.2; 0.3)	0.2 (0.2; 0.3)
12–13 years	4.1 (3.6; 4.5)	3.1 (3.0; 3.3)	1.4 (1.3; 1.5)	1.7 (1.7; 1.8)	5.1 (4.6; 5.5)	6.1 (6.0; 6.3)	0.3 (0.0; 0.5)	0.2 (0.2; 0.3)	0.3 (0.1; 0.4)	0.3 (0.2; 0.3)
14 years or more	4.9 (4.6; 5.2)	4.2 (4.0; 4.4)	1.6 (1.5; 1.6)	1.8 (1.7; 1.8)	4.5 (4.2; 4.7)	4.4 (4.3; 4.6)	0.2 (0.2; 0.3)	0.5 (0.4; 0.5)	0.3 (0.2; 0.4)	0.4 (0.3; 0.4)
**Wealth quintiles**										
Q1–Poorest	3.5 (3.3; 3.7)	3.0 (2.8; 3.1)	1.5 (1.4; 1.5)	1.7 (1.7; 1.8)	8.7 (8.3; 9.0)	7.7 (7.5; 7.9)	0.1 (0.1; 0.1)	0.2 (0.2; 0.3)	0.3 (0.2; 0.4)	0.4 (0.3; 0.4)
Q2	3.5 (3.3; 3.7)	3.1 (3.0; 3.3)	1.5 (1.5; 1.5)	1.8 (1.7; 1.8)	7.8 (7.5; 8.1)	7.0 (6.8; 7.2)	0.1 (0.0; 0.1)	0.2 (0.1; 0.2)	0.2 (0.2; 0.3)	0.3 (0.3; 0.3)
Q3	3.9 (3.7; 4.1)	3.4 (3.2; 3.5)	1.5 (1.5; 1.6)	1.8 (1.8; 1.9)	7.3 (7.0; 7.6)	6.6 (6.4; 6.8)	0.1 (0.1; 0.1)	0.1 (0.1; 0.2)	0.2 (0.2; 0.3)	0.3 (0.2; 0.4)
Q4	3.9 (3.7; 4.1)	3.5 (3.3; 3.7)	1.6 (1.6; 1.7)	1.9 (1.8; 1.9)	7.0 (6.7; 7.3)	6.3 (6.0; 6.5)	0.1 (0.1; 0.2)	0.2 (0.1; 0.2)	0.2 (0.1; 0.2)	0.2 (0.2; 0.3)
Q5–Wealthiest	4.7 (4.5; 5.0)	4.0 (3.8; 4.2)	1.6 (1.5; 1.6)	1.8 (1.7; 1.9)	5.4 (5.1; 5.7)	5.1 (4.9; 5.4)	0.2 (0.2; 0.3)	0.4 (0.3; 0.5)	0.3 (0.2; 0.4)	0.3 (0.2; 0.4)
**Total**	4.0 (3.8; 4.1)	3.4 (3.3; 3.5)	1.5 (1.5; 1.6)	1.8 (1.8; 1.8)	7.1 (7.0; 7.3)	6.5 (6.4; 6.6)	0.1 (0.1; 0.2)	0.2 (0.2; 0.2)	0.3 (0.2; 0.3)	0.3 (0.3; 0.3)

For the subgroups of healthy foods, inequality measures are presented in Table 3. The estimates stratified by sex indicate that women consumed less calories from pulses and more from fruits and vegetables than men in both years. Absolute inequalities between males and females have changed between surveys for pulses, with a reduction of the difference from −1.6 p.p. to −1.3 p.p., whereas an increase in the difference was observed for vegetables, from 0.1 p.p. to 0.2 p.p.

**Table 3:** Inequality measures for the set of plant-based natural or minimally processed foods subgroups in 2008 and 2017.

	**Difference** ^a^				**Slope index of inequality** ^b^			
	**2008**		**2017**		**2008**		**2017**	
	**Estimate**	**95%CI**	**Estimate**	**95%CI**	**Estimate**	**95%CI**	**Estimate**	**95%CI**
**Fruits**								
Sex	1.2	1.0; 1.3	1.3	1.1; 1.4	-	-	-	-
Area	−0.1	−0.4; 0.2	−0.2	−0.4; 0.0	-	-	-	-
Education	-	-	-	-	0.7	0.4; 1.1	0.5	0.3; 0.8
Wealth quintiles	-	-	-	-	1.5	1.1; 1.9	1.2	0.9; 1.5
**Vegetables**								
Sex	0.1	0.1; 0.1	0.2	0.1; 0.2^c^	-	-	-	-
Area	-0.1	−0.1; 0.0	0.0	−0.1; 0.0	-	-	-	-
Education	-	-	-	-	−0.2	−0.3; −0.1	−0.2	−0.3; −0.2
Wealth quintiles	-	-	-	-	0.2	0.1; 0.3	0.1	0.0; 0.2
**Pulses**								
Sex	−1.6	−1.7; −1.4	−1.3	−1.4; −1.1^c^	-	-	-	-
Area	1.5	1.1; 1.9	1.4	1.1; 1.7	-	-	-	-
Education	-	-	-	-	−4.7	−5.1; −4.3	−4.0	−4.3; −3.7
Wealth quintiles	-	-	-	-	−3.7	−4.3; −3.2	−2.9	−3.3; −2.5^c^
**Nuts and seeds**								
Sex	0.0	0.0; 0.1	0.1	0.0; 0.1	-	-	-	-
Area	0.0	−0.1; 0.0	0.0	−0.1; 0.0	-	-	-	-
Education	-	-	-	-	0.2	0.1; 0.3	0.3	0.3; 0.4
Wealth quintiles	-	-	-	-	0.2	0.1; 0.3	0.2	0.0; 0.3
**Whole grains**								
Sex	0.0	0.0; 0.1	0.1	0.0; 0.1	-	-	-	-
Area	0.1	0.0; 0.2	0.1	0.0; 0.2	-	-	-	-
Education	-	-	-	-	0.0	−0.1; 0.1	0.1	0.0; 0.2
Wealth quintiles	-	-	-	-	0.0	−0.1; 0.1	−0.1	−0.2; 0.0

a Difference (female—male, rural—urban) estimated for binary stratifiers.

b Slope index of inequality estimated for ordinal stratifiers.

c Evidence of differences in the estimates between the 2008 and the 2017 surveys was considered based upon the non-overlapping of confidence intervals.

The consumption of the fruits increased as the education and wealth level increased in both surveys while the opposite was observed for the consumption of pulses in both surveys. In addition, consumption of pulses was higher for those living in rural area. No significant changes in the inequality measures between 2008 and 2017 were observed for area of residence and educational levels. Consumption of pulses decreased according to wealth quintiles and the estimate of wealth-based inequalities, measured by the SII, decreased from −3.7 p.p. to −2.9 p.p.

## Discussion

Our analysis showed a small but significant increase of 1.3 p.p. in the consumption of ultra-processed foods and a concomitant reduction of 1.2 p.p. in the contribution of the set of plant-based natural or minimally processed foods to the total diet between 2008 and 2017 among Brazilian adults. We observed that the urban population and those in the wealthier group and with higher education levels presented higher consumption of ultra-processed foods and lower consumption of plant-based natural or minimally processed foods in both survey years, whereas no expressive differences were observed by sex. These differences were much more prominent for the consumption of ultra-processed foods. However, over the 10-year period, there was an overall reduction of the socioeconomic inequalities and, therefore, a deterioration of the dietary quality of the more disadvantaged groups.

Ultra-processed foods have always been promoted and advertised incessantly with “seductive” messages that may led people to believe they are superior to traditional dishes like rice and beans and that they will make people happier (17). On the other hand, contrary to what happens in Global North countries, these foods have always been more expensive and more accessible in urbanized areas (21). As a consequence, the consumption of ultra-processed foods has been higher among wealthiest people in Brazil. However, reductions in the inequalities in their consumption can be explained by the expansion of the access to unhealthy foods by lower socioeconomic classes, which may be due to the reduction in their prices, the expansion of their offer in the most diverse purchase places and the increasing penetration of transnational food companies in more remote and rural areas of the country (22–24). Analysis of data from the National System of Consumer Price Indexes shows that, although ultra-processed foods are still more expensive than natural or minimally processed foods and culinary ingredients, since the early 2000s, an inversion trend in the prices has been observed (24). The price of natural or minimally processed foods and culinary ingredients increased continuously from 2003 to 2017 (from R$ 4.43/kg to R$ 4.70/kg). On the other hand, the price of ultra-processed foods showed an opposite trend, decreasing from R$ 7.31/kg to R$ 6.67/kg in the same period. In addition, the relative price of healthy foods in relation to unhealthy foods increased over the period, from 53% in 1995 to 70% in 2017 (24). More recent data indicate that, since 2020, this trend has been accelerating and that, soon, ultra-processed foods will be cheaper than other foods (25).

In recent years, there has also been an increase in the purchase of foods in supermarket chains and previous studies indicate that they offer a greater concentration of ultra-processed foods compared to other traditional shopping sites. In 2008-2009, there was a direct and significant relationship between the participation of supermarkets in total food acquisition and the consumption of ultra-processed foods by the Brazilian population (26). Specific marketing of ultra-processed foods to lower-income communities has also helped to accelerate their consumption growth in poorer segments of Brazilian society. Some of the “Big food” companies, for example, have implemented “popular positioning products” projects, which are targeted at low-income consumers and drive door-to-door sales of ultra-processed foods on the outskirts of several Brazilian cities, on trains and subway stations, in retail chains that sell electronics and appliances, and also in “floating supermarkets” that take these products to remote Amazonian communities (27).

Our results also showed that there are different patterns in the distribution of consumption of healthy food subgroups: while fruits are more consumed by people of higher socioeconomic conditions, the opposite is observed regarding the consumption of beans. On the other hand, our results also showed that the drop in the consumption of healthy foods is mainly driven by the trend of decreasing consumption of beans (−0.7 p.p.). This was accompanied by a reduction in wealth inequality, which means an even more accentuated decline in the consumption of this food group among the poorest. These data are extremely worrying considering that beans, combined with rice, are one of the most traditional foods in the Brazilian diet, making up a healthy and sustainable meal, in addition to being relatively cheap (17). In 2008, for example, the average price of beans was almost 60% lower than the average price of the entire set of natural or minimally processed foods (27) and the price of the group of pulses and cereals has not increased since 1995 (24). On the other hand, culinary preparations made of beans require more time and better cooking skills, which may be associated with the downward trend in their consumption. A recent study with data on household food purchases by Brazilian families showed that, unlike plant-based natural or minimally processed foods, unprocessed meats, especially red meats, followed the upward trend of ultra-processed foods in all income strata (28).

Telephone-based study carried out in Brazilian capitals only evaluated the frequency of consumption of some food consumption markers and also showed that the consumption of fruits was more prevalent among the more educated citizens, while beans were mostly consumed by the less educated. Oppositely, the consumption of soft drinks (an ultra-processed food) was more frequent among those in the intermediate groups of education (9). As far as we know, our study is the first evaluating inequalities in food consumption with a representative sample of the entire country, from both rural and urban areas, in which the food consumption was evaluated using a very comprehensive method of data collection—two 24-h food records in 2008 and two 24-h food recalls in 2017—and that evaluated a broad group of socioeconomic measures.

Our data raise a debate about the complexity of discussing socioeconomic inequalities in food consumption. While, for some important health outcomes, the reduction of the difference between groups defined by social characteristics represents better living conditions for all, this does not always seem true when it comes to food intake. The nutrition transition, characterized by the rise of a globalized food system dominated by the agribusiness and the “Big Food” companies, benefits from the increase in the purchasing power of populations while bringing as side effects chronic diseases that affect the poorer most intensely. This phenomenon is highly marked in middle-income countries like Brazil. It is important to point out that until 2014, Brazil had been showing a fundamental economic growth and improvement in the social conditions of its population, which may have reflected in the reduction of inequality in food consumption between the richest and poorest. After that, the country began suffering an economic recession and a political crisis, both of which exacerbated by the Covid-19 pandemic. Considering that this scenario significantly increased the food insecurity levels and worsened the nutrition conditions of the Brazilian population (29), our study can serve as a good “baseline” to assess the post-pandemic evolution of these indicators. It is necessary to politicize the debate on inequalities, advocating policies that not only increase the purchasing power of the population, but that protect people from unhealthy food environments, which includes the massive advertising of ultra-processed foods, and guarantee the right to nutritious, affordable and sustainably-produced foods.

We are aware that our study has some limitations, mainly related to potential biases inherent to dietary surveys, including the possibility of under or overestimation of the consumption of certain food groups; differences between real cooking recipes and standardized recipes; and differences between the real nutritional composition of the foods consumed and that from the nutritional composition table. However, data collection instruments were pre-tested and validated and inconsistent records were excluded and replaced with imputed values, after quality control. In addition, the food nutritional composition table used to calculate energy was built specifically for the Brazilian population, and food consumption estimates were adjusted by the Multiple Source Method to account for the variability of the 2 days of consumption. Finally, it is important to highlight that different methods were applied to collect food consumption information in the two surveys (food records in 2008 and 24 h food recalls in 2017). Nevertheless, a previous publication showed that these changes, in general, had little effect on the estimation of diet composition, allowing comparison between the two databases after harmonization strategies (databases were made compatible and the information from the 2008 survey was reanalyzed using the same food composition table used in the 2017 survey) (15). On the other hand, the strengths of this study include the rigorously probabilistic nature of the samples analyzed and the national representativeness, ensured by the inclusion of more than 70 thousand people living in urban and rural areas from all the regions of the country, and also the availability of a database with more than 1,800 food items. Besides that, the use of absolute, relative and complex measures of inequality brings a robustness to the estimates and conclusions. The slope index of inequality, for example, measures the absolute inequality of the indicator between the most privileged individuals and the less privileged individuals, taking into consideration the entire distribution of the stratification variable (30).

In conclusion, our results showed marked socioeconomic inequalities in food consumption among Brazilian adults, mainly in the ultra-processed food consumption. However, contrary to expectations, a reduction of the socioeconomic inequalities may lead to the overall deterioration of the dietary quality of the more disadvantaged groups.

## Data availability statement

Publicly available datasets were analyzed in this study. This data can be found here: https://www.ibge.gov.br/.

## Ethics statement

Ethical review and approval was not required for the study on human participants in accordance with the local legislation and institutional requirements. Written informed consent for participation was not required for this study in accordance with the national legislation and the institutional requirements.

## Author contributions

JC and AW run data analyses. All authors were responsible for planning the analyses, interpreting the results, and writing the paper. All authors contributed to the article and approved the submitted version.

## References

[1] Vandevijvere S Jaacks LM Monteiro CA Moubarac JC Girling-Butcher M Lee AC. Global trends in ultraprocessed food and drink product sales and their association with adult body mass index trajectories. Obes Rev. (2019) 20 (Suppl. 2):10–9. 10.1111/obr.12860 31099480

[2] Monteiro CA Moubarac JC Cannon G Ng SW Popkin B. Ultra-processed products are becoming dominant in the global food system. Obes Rev. (2013) 14 (Suppl. 2):21–8. 10.1111/obr.12107 24102801

[3] Monteiro CA Cannon G Lawrence M Costa Louzada ML Pereira Machado P. Ultra-Processed Foods, Diet Quality, and Health Using the NOVA Classification System. Rome: FAO (2019).

[4] Willett W Rockström J Loken B Springmann M Lang T Vermeulen S. Food in the Anthropocene: the EAT-Lancet Commission on healthy diets from sustainable food systems. Lancet. (2019) 393:447–92. 10.1016/S0140-6736(18)31788-4 30660336

[5] Swinburn BA Kraak VI Allender S Atkins VJ Baker PI Bogard JR. The global syndemic of obesity, undernutrition, and climate change: the lancet commission report. Lancet. (2019) 393:791–846. 10.1016/S0140-6736(18)32822-8 30700377

[6] Otero G Gürcan EC Pechlaner G Liberman G. Food security, obesity, and inequality: Measuring the risk of exposure to the neoliberal diet. J Agra Change. (2018) 18:536–54. 10.1111/joac.12252

[7] Honório OS Pessoa MC Gratão LHA Rocha LL de Castro IRR Canella DS. Social inequalities in the surrounding areas of food deserts and food swamps in a Brazilian metropolis. Int J Equity Health. (2021) 20:168. 10.1186/s12939-021-01501-7 34289857 PMC8293554

[8] Dulgheroff PT da Silva LS Rinaldi AEM. Educational disparities in hypertension, diabetes, obesity and smoking in Brazil: a trend analysis of 578 977 adults from a national survey, 2007–2018. BMJ Open. (2021) 0:e046154. 10.1136/bmjopen-2020-046154 34281920 PMC8291309

[9] Crepaldi BVC Okada LM Rauber F Levy RB Azeredo CM. Social inequality in food consumption between 2008 and 2019 in Brazil. Public Health Nutr. (2022) 25:214–24. 10.1017/S1368980021002950 34407905 PMC8883783

[10] Mayén A-L Marques-Vidal P Paccaud F Bovet P Stringhini S. (2014) Socioeconomic determinants of dietary patterns in low- and middle-income countries: a systematic review. Am J Clin Nutr. (2014) 100:1520–31. 10.3945/ajcn.114.089029 25411287

[11] Hosseinpoor AR Bergen N Kunst A Harper S Guthold R Rekve D. Socioeconomic inequalities in risk factors for non communicable diseases in low-income and middle-income countries: results from the World Health Survey. BMC Public Health. (2012) 12:912. 10.1186/1471-2458-12-912 23102008 PMC3507902

[12] Lock K Pomerleau J Causer L McKee M. Low Fruit and Vegetable Consumption. Comparative Quantification of Health Risks: Global and Regional Burden of Disease Attributable to Selected Major Risk Factors. Ezzati M Lopez AD Rodgers A Murray CJL. Geneva: World Health Organization (2004). p. 597–728.

[13] Pesquisa de. orçamentos familiares 2017-2018: análise do consumo alimentar pessoal no Brasil / IBGE, Coordenação de Trabalho e Rendimento. Rio de Janeiro: IBGE (2020).

[14] Pesquisa de. orçamentos familiares 2008-2009: análise do consumo alimentar pessoal no Brasil / IBGE, Coordenação de Trabalho e Rendimento. Rio de Janeiro: IBGE (2011).

[15] Rodrigues RM Carli E Araújo MC Verly Junior E Marchioni DML Bezerra IN. Limitations in the comparison of the Brazilian National Dietary Surveys of 2008-2009 and 2017-2018. Rev Saude Publica. (2021) 55 (Suppl. 1): 3s. English, Portuguese. 10.11606/s1518-8787.2021055003365 34910053 PMC9586432

[16] Monteiro CA Cannon G Levy RB Moubarac JC Louzada ML Rauber F. Ultra-processed foods: what they are and how to identify them. Public Health Nutr. (2019) 22:936–41. 10.1017/S1368980018003762 30744710 PMC10260459

[17] Brazilian Ministry of Health. Dietary Guidelines for the Brazilian Population. Brasília: Brazilian Ministry of Health (2014).

[18] Food and Agriculture Organization. Healthy and Sustainable Diets. Rome: FAO (2019).

[19] Rutstein Shea O Johnson K. The DHS Wealth Index. Maryland: ORC Macro (2004).

[20] Silva ICMD Restrepo-Mendez MC Costa JC Ewerling F Hellwig F Ferreira LZ. Measurement of social inequalities in health: concepts and methodological approaches in the Brazilian context. Epidemiol Serv Saude. (2018) 27:e000100017. 10.5123/S1679-49742018000100017 29513856 PMC7705122

[21] Moubarac JC Claro RM Baraldi LG Levy RB Martins AP Cannon G. International differences in cost and consumption of ready-to-consume food and drink products: United Kingdom and Brazil, 2008-2009. Glob Public Health. (2013) 8:845–56. 10.1080/17441692.2013.796401 23734735

[22] Monteiro CA Cannon G. The impact of transnational “big food” companies on the South: a view from Brazil. PLoS Med. (2012) 9:e1001252. 10.1371/journal.pmed.1001252 22802732 PMC3389019

[23] Monteiro CA Cannon G. The role of the transnational ultra-processed food industry in the pandemic of obesity and its associated diseases: problems and solutions. World Nutrition. (2019) 10:89–99. 10.26596/wn.201910189-99

[24] Maia EG Dos Passos CM Levy RB Bortoletto Martins AP Mais LA Claro RM. What to expect from the price of healthy and unhealthy foods over time? The case from Brazil. Public Health Nutr. (2020) 23:579–88. 10.1017/S1368980019003586 31937385 PMC7058424

[25] Johns P Albiero MS. Dinâmica e diferenças dos preços dos alimentos no Brasil [livro eletrônico]. São Paulo: ACT Promoção da Saúde (2021).

[26] Machado PP Claro RM Martins APB Costa JC Levy RB. Is food store type associated with the consumption of ultra-processed food and drink products in Brazil? Public Health Nutr. (2018) 21:201–9. 10.1017/S1368980017001410 28756782 PMC10260784

[27] Claro RM Maia EG Costa BV Diniz DP. Preço dos alimentos no Brasil: prefira preparações culinárias a alimentos ultraprocessados [Food prices in Brazil: prefer cooking to ultra-processed foods]. Cad Saude Publica. (2016) 32:e00104715. Portuguese. 10.1590/0102-311X00104715 27580234

[28] Levy RB Andrade GC Cruz GLD Rauber F Louzada MLDC Claro RM. Three decades of household food availability according to NOVA - Brazil, 1987–2018. Rev Saude Publica. (2022) 56:75. English, Portuguese. 10.11606/s1518-8787.2022056004570 35946675 PMC9388064

[29] Instituto Brasileiro de Geografia e Estatística Coordenação de Trabalho e Rendimento. Pesquisa de Orçamentos Familiares 2017-2018: análise da segurança alimentar no Brasil. Rio de Janeiro: IBGE (2020).

[30] da Silva ICM Restrepo-Mendez MC Costa JC Ewerling F Hellwig F Ferreira LZ et al. Mensuração de desigualdades sociais em saúde: conceitos e abordagens metodológicas no contexto brasileiro. Epidemiol Serv Saúde. (2018) 27:1–12. 10.5123/S1679-49742018000100017 PMC7705122 29513856

